# Supplementary material for: Megalurothrips usitatus Directly Causes the Black-Heads and Black-Tail Symptoms of Cowpea along with the Production of Insect-Resistance Flavonoids
Source: Plants (Basel). 2023 Nov 15;12(22):3865. doi: 10.3390/plants12223865 (PMC10675644; doi:10.3390/plants12223865)
Supplement: Supplementary file 1 [file plants-12-03865-s001.zip › plants-2689969-supplementary.pdf]

Megalurothrips usitatus directly causes the black-heads and black-tail symptoms of cowpea along with the production of insect-resistance flavonoids

Yunchuan He [1, 2], Yang Gao [1, 2], Hainuo Hong [1, 2], Jiamei Geng [1, 2], Qiulin Chen [1, 2], Ying Zhou [1, *], Zengrong Zhu [1, 2]

[1] Hainan Institute, Zhejiang University, Yazhou District, Sanya 572025, China
[2] State Key Laboratory of Rice Biology, Institute of Insect Sciences, Zhejiang University, Hangzhou, 310058, China
* Correspondence: yzhyzb@zju.edu.cn

Figure S1 Laboratory verification formation of black-head and black-tail symptoms

Healthy pods with the same growing conditions were selected. The pods were cut about 2 cm, then the cut pods were placed into cap of 50 ml centrifuge tube cap, and parafilm sealing, a total of 30. Finally, the cap is placed in the incubator (BIC-250, Shanghai Boxun Medical Biological Instrument Co. Ltd., China) at a temperature of 26 ± 0.5°C, 60 ± 5% RH, and a photoperiod of 14:10 (L:D) h. Among them, no less than 100 adult female thrips that were 3-days old were introduced into T treatment. The formation of black-head and black-tail symptoms were recorded every day with a Canon camera (EOS 5D Mark IV) (Figure S1).

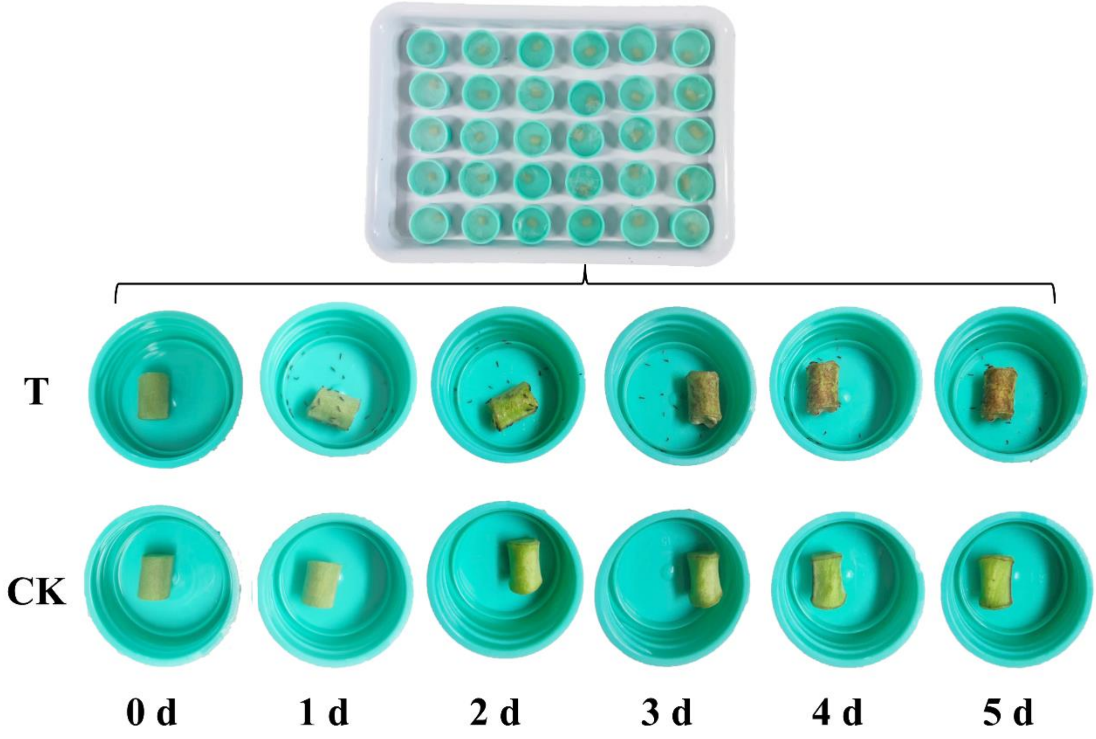

Figure S1 Laboratory verification formation of black-head and black-tail symptoms
CK: The pod with untreated by M. usitatus. T: The pod with treated by *M. usitatus*.

Figure S2 Thrip insecticide bioassay system (TIBS)

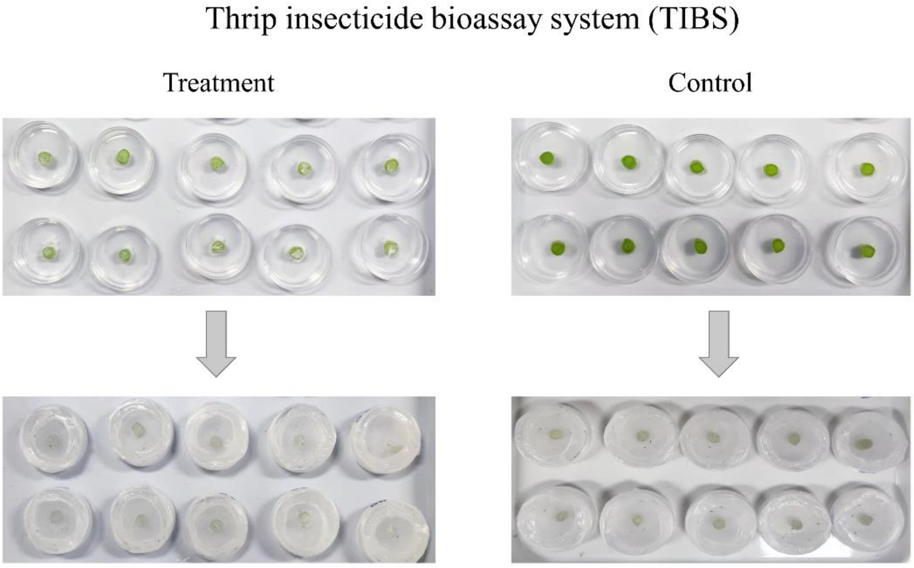

Figure S2 Thrip insecticide bioassay system (TIBS)

Treatment：4 mg/ml 7,4'-Dihydroxyflavone, 4 mg/ml coumestrol. Control: 4% DMSO.

Table S1 Results of differential secondary metabolites

Index	Formula	Compounds	Class	VIP	*P*-value	Log2FC	Type
Hmtn001302	$C_{13}H_{16}O_8$	Glucosyloxybenzoic acid	Phenolic acids	1.09	0.01	1.68	up
pmb3107	$C_{15}H_{20}O_{10}$	Glucosyringic Acid	Phenolic acids	1.19	0.00	4.18	up
Lmhn003373	$C_{11}H_{10}O_7$	4-Hydroxybenzoylmalic acid	Phenolic acids	1.19	0.02	6.14	up
mws1212	$C_{11}H_{12}O_4$	Ferulic acid methyl ester	Phenolic acids	1.14	0.01	1.78	up
MWS1846	$C_8H_8O_3$	Phenoxyacetic acid	Phenolic acids	1.19	0.00	6.99	up
Hmhn003067	$C_{15}H_{18}O_8$	Phenylpropionic acid-O-β-D-glucopyranoside	Phenolic acids	1.19	0.00	2.16	up
mws4102	$C_{21}H_{18}O_6$	Isoglycyrol	Lignans and Coumarins	1.19	0.00	14.50	up
Hmyp003337	$C_{18}H_{20}O_6$	Tabaisocoumarin D	Others	1.19	0.00	15.75	up
mws4002	$C_8H_{11}NO_2$	Dopamine	Alkaloids	1.19	0.01	-11.96	down
Jmzn006005	$C_{10}H_{10}O_3$	3,4-Methylenedioxy cinnamyl alcohol	Lignans and Coumarins	1.07	0.20	-3.10	down
Lmdn007639	$C_{15}H1_2O_4$	3,9-Dihydroxypterocarpan	Flavonoids	1.19	0.02	5.97	up
Lmjp002906	$C_{22}H_{22}O_{12}$	Rhamnetin-3-O-Glucoside*	Flavonoids	1.19	0.01	2.65	up
Lmmp002080	$C_{11}H_{16}N_2O$	N-(4-Aminobutyl)benzamide	Alkaloids	1.19	0.01	15.48	up
Zmdp008296	$C_{20}H_{18}O_5$	6-prenylapigenin	Others	1.20	0.00	14.64	up
Lmmn000774	$C_{15}H_{20}O_9$	Dihydrocaffeoylglucose	Phenolic acids	1.19	0.00	6.41	up
Hmbn004734	$C_{29}H_{44}O_4$	30-Norhederagenin	Terpenoids	1.17	0.02	2.01	up
mws1405	$C_{15}H_8O_5$	Coumestrol	Lignans and Coumarins	1.19	0.01	17.28	up
pmb3068	$C_{16}H_{18}O_8$	1-O-p-Coumaroylquinic acid	Phenolic acids	1.19	0.01	-16.52	down
MA10107783	$C_{10}H_8O_5$	3-[(1-Carboxyvinyl)oxy]benzoic acid	Phenolic acids	1.14	0.00	4.51	up
mws1173	$C_{15}H_{12}O_5$	Garbanzol	Flavonoids	1.19	0.10	16.12	up
mws0093	$C_{10}H_{12}O_3$	Coniferyl alcohol	Phenolic acids	1.11	0.04	3.95	up
Lmdn006025	$C_{15}H_{12}O_6$	2-Hydroxy-2,3-dihydrogenistein	Flavonoids	1.19	0.07	18.08	up
mws1521	$C_{13}H_{18}O_7$	Salicin	Phenolic acids	1.16	0.01	1.30	up
pmb0782	$C_5H_{11}N$	Piperidine	Alkaloids	1.19	0.01	2.37	up

pmb3074	$C_{16}H_{18}O_8$	5-O-p-Coumaroylquinic acid*	Phenolic acids	1.01	0.14	-1.41	down
Lmzn006284	$C_{30}H_{48}O_4$	2α-Hydroxyursolic acid	Terpenoids	1.19	0.01	-15.10	down
pmn001419	$C_{15}H_{18}O_8$	1-O-p-Coumaroyl-β-D-glucose*	Phenolic acids	1.19	0.00	2.00	up
HJAP064	$C_{25}H_{24}O_{15}$	Isorhamnetin-3-O-(6"-malonyl)glucoside	Flavonoids	1.09	0.08	2.28	up
Lmdn009099	$C_{20}H_{20}O_6$	Coniferyl ferulate	Phenolic acids	1.19	0.03	7.73	up
Lmhn006533	$C_{17}H_{24}O_9$	Awsoniaside B	Phenolic acids	1.19	0.00	13.46	up
pmn001423	$C_{19}H_{30}O_8$	Roseoside	Others	1.18	0.00	2.29	up
mws0005	$C_{10}H_{12}N_2$	Tryptamine	Alkaloids	1.19	0.13	13.06	up
MWSHY0147	$C_{15}H_{10}O_4$	Daidzein	Flavonoids	1.19	0.04	6.49	up
MWSHY0072	$C_{19}H_{20}O_5$	Methylophiopogonanone B	Flavonoids	1.06	0.01	-2.07	down
mws1350	$C_9H_{10}O_4$	Syringaldehyde; 4-Hydroxy-3,5-Dimethoxybenzaldehyde	Phenolic acids	1.19	0.01	4.82	up
NK10253223	$C_8H_9NO_3$	2-Amino-3-methoxybenzoic acid	Phenolic acids	1.19	0.00	-2.43	down
Wmkn004295	$C_{26}H_{30}O_6$	Kuraridin	Flavonoids	1.19	0.02	12.90	up
mws1200	$C_{10}H_{10}O_3$	Trans-4-Hydroxycinnamic Acid Methyl Ester	Phenolic acids	1.06	0.21	-3.06	down
mws0749	$C_7H_6O_3$	4-Hydroxybenzoic acid	Phenolic acids	1.19	0.01	3.22	up
mws0943	$C_7H_8N_2O_2$	1,4-Dihydro-1-Methyl-4-oxo-3-pyridinecarboxamide	Alkaloids	1.19	0.01	3.57	up
mws0097	$C_{20}H_{22}O_6$	Pinoresinol	Lignans and Coumarins	1.20	0.00	15.13	up
Xmbp001692	$C_7H_6O_5$	2,4,6-Trihydroxybenzoic acid	Phenolic acids	1.19	0.00	14.11	up
Lmhn004976	$C_{16}H_{12}O_6$	Aracarpene 2	Flavonoids	1.19	0.00	7.06	up
HJN055	$C_{21}H_{24}O_{10}$	Dihydrocharcone-4'-O-glucoside	Flavonoids	1.08	0.05	1.25	up
MWSmce675	$C_{12}H_{16}O_7$	Arbutin	Phenolic acids	1.19	0.00	3.89	up
mws0145	$C_8H_8O_3$	O-Anisic acid (2-Methoxybenzoic acid)	Phenolic acids	1.19	0.00	6.98	up

Zmpn002440	$C_{15}H_{12}O_6$	3,4,2',4',6'-Pentahydroxychalcone	Flavonoids	1.20	0.00	18.50	up
mws0914	$C_{15}H_{12}O_5$	3,5,7-Trihydroxyflavanone (Pinobanksin)	Flavonoids	1.19	0.01	6.58	up
pmb0686	$C_{24}H_{24}O_{14}$	Eriodictyol-7-O-(6"-malonyl)glucoside	Flavonoids	1.19	0.01	12.76	up
mws0058	$C_{16}H_{12}O_6$	Diosmetin (5,7,3'-Trihydroxy-4'-methoxyflavone)	Flavonoids	1.19	0.00	6.85	up
pmb0774	$C_{10}H_{12}N_2O$	N-Hydroxytryptamine	Alkaloids	1.19	0.00	11.98	up
mws0983	$C_{20}H_{39}NO_2$	N-Oleoylethanolamine	Alkaloids	1.05	0.03	1.34	up
Hmjn003948	$C_{30}H_{48}O_5$	Madasiatic acid	Terpenoids	1.19	0.02	14.00	up
Lmyn006227	$C_{15}H_{10}O_5$	Galangin (3,5,7-Trihydroxyflavone)	Flavonoids	1.19	0.01	7.21	up
Zmhn003082	$C_{15}H_{18}O_{10}$	5-O-Galloyl-methyl quinine ester	Phenolic acids	1.17	0.04	-2.43	down
Smcp000882	$C_{15}H_{21}NO_7$	N-benzoyl-2-aminoethyl-β-D-glucopyranoside	Alkaloids	1.19	0.10	-13.37	down
Lssp210056	$C_{21}H_{20}O_{12}$	Quercetin3-O-galactoside	Flavonoids	1.18	0.00	-1.86	down
pmp000351	$C_{20}H_{18}O_5$	Eurycarpin A	Flavonoids	1.19	0.17	13.66	up
mws0063	$C_{15}H_{10}O_5$	Genistein	Flavonoids	1.18	0.04	5.52	up
Hmlp005105	$C_{16}H_{12}O_5$	3,7-dihydroxy-4'-methoxyflavone	Flavonoids	1.19	0.13	15.79	up
MWSHY0079	$C_{21}H_{22}O_9$	Liquiritigenin-4'-O-Glucoside (Liquiritin)	Flavonoids	1.18	0.00	3.30	up
pme0376	$C_{15}H_{12}O_5$	Naringenin (5,7,4'-Trihydroxyflavanone)	Flavonoids	1.19	0.01	7.00	up
MWS2058	$C_8H_8O_3$	3-Methylsalicylic Acid	Phenolic acids	1.19	0.00	12.30	up
mws0908	$C_{16}H_{12}O_5$	Glycitein	Flavonoids	1.19	0.17	16.05	up
Lskp211345	$C_{21}H_{32}O_4$	Neocaesalpin E	Terpenoids	1.02	0.08	1.66	up
MWSHY0162	$C_{27}H_{30}O_{17}$	Quercetin-3-O-sophoroside (Baimaside)	Flavonoids	1.15	0.01	-1.22	down
Zmhn000892	$C_{13}H_{18}O_8$	4-O-Glucosyl-3,4-dihydroxybenzyl alcohol	Phenolic acids	1.18	0.01	1.40	up
Lmhn003801	$C_{25}H_{24}O_{13}$	Feruloylsinapoyltartaric acid	Phenolic acids	1.15	0.00	1.62	up

mws0044	$C_{15}H_{12}O_7$	Taxifolin(Dihydroquercetin)	Flavonoids	1.18	0.00	8.27	up
mws0906	$C_{16}H_{22}O_8$	Coniferin	Phenolic acids	1.19	0.00	2.73	up
MWSmce052	$C_{30}H_{48}O_3$	3-Epiursolic acid	Terpenoids	1.19	0.00	6.07	up
pmp000384	$C_{21}H_{22}O_9$	Isoliquiritin	Flavonoids	1.18	0.00	2.71	up
Lmmp003817	$C_{24}H_{22}O_{14}$	Kaempferol-3-O-(6"-malonyl)glucoside*	Flavonoids	1.09	0.01	1.17	up
mws0444	$C_7H_7NO_3$	3-Aminosalicylic acid	Phenolic acids	1.19	0.02	-11.86	down
Lmsn002288	$C_{21}H_{28}O_{14}$	1-O-Caffeoyl-(6-O-glucosyl)-β-D-glucose	Phenolic acids	1.19	0.01	2.64	up
mws0066	$C_{16}H_{12}O_7$	Isorhamnetin; 3'-Methoxy-3,4',5,7-Tetrahydroxyflavone	Flavonoids	1.04	0.03	1.00	up
mws0458	$C_8H_8O_3$	Vanillin; 4-Hydroxy-3-Methoxybenzaldehyde	Phenolic acids	1.18	0.04	4.74	up
mws1329	$C_{21}H_{20}O_{12}$	Quercetin-7-O-glucoside	Flavonoids	1.17	0.00	-1.29	down
Zmxp002867	$C_{28}H_{32}O_{16}$	Chrysoeriol-5,7-di-O-glucoside	Flavonoids	1.18	0.01	2.69	up
Lmqn001932	$C_{28}H_{36}O_{13}$	Syringaresinol-4'-O-glucoside	Lignans and Coumarins	1.20	0.00	15.12	up
pme3217	$C_{15}H_{12}O_4$	Isoliquiritigenin	Flavonoids	1.19	0.02	6.37	up
Smpn009230	$C_{30}H_{48}O_5$	2α,3α,23-trihydroxyolean-12-en-28-oic acid	Terpenoids	1.20	0.00	13.90	up
Wmkn003797	$C_{21}H_{22}O_6$	(2R,3R)-8-Prenyl-7,4'-dihydroxy-5-methoxydihydroflavonol	Flavonoids	1.19	0.01	11.43	up
Lmbp000728	C_8H_8O	(S)-2-Phenyloxirane	Phenolic acids	1.19	0.00	6.42	up
pmp001287	C_8H_9N	N-Benzylmethylene isomethylamine	Alkaloids	1.12	0.02	1.03	up
MWSHY0104	$C_{21}H_{20}O_{11}$	Luteolin-7-O-glucoside (Cynaroside)	Flavonoids	1.02	0.02	1.11	up
mws0704	$C_2H_8NO_4P$	O-Phosphorylethanolamine	Alkaloids	1.18	0.00	2.71	up
Lmjp003044	$C_{22}H_{22}O_{12}$	Isorhamnetin-3-O-Glucoside*	Flavonoids	1.19	0.00	2.64	up
Lssp210058	$C_{27}H_{30}O_{16}$	Quercetin-3-O-robinobioside*	Flavonoids	1.17	0.03	-2.43	down

Lhmp122214	$C_{16}H_{16}O_5$	5,6-dihydroxy-2-methoxy-5-(1-phenyl-2-propenyl)-2-cyclohexene-1,4-dione	Others	1.19	0.02	13.63	up
mws4057	$C_{15}H_{10}O_4$	7,4'-Dihydroxyflavone	Flavonoids	1.20	0.00	17.23	up
Lmsp013116	$C_{20}H_{32}O_3$	Ent-16α,17-Dihydroxykauran-2-one	Terpenoids	1.19	0.01	14.80	up
HJKP000649	C_8H_9NO	N-benzylformamide	Alkaloids	1.18	0.01	1.19	up
MWSHY0069	$C_{16}H_{12}O_6$	Hispidulin (5,7,4'-Trihydroxy-6-methoxyflavone)	Flavonoids	1.19	0.00	6.71	up
Jmgn005927	$C_{15}H_{12}O_6$	2-hydroxynaringenin	Flavonoids	1.19	0.08	7.79	up
Lmhn004960	$C_{22}H_{18}O_{10}$	7-O-Galloyltricetiflavan	Flavonoids	1.18	0.17	-14.55	down
pmb0069	C_7H_7NO	Benzamide	Phenolic acids	1.20	0.00	13.48	up
Wmkn003942	$C_{21}H_{22}O_6$	2'-HydroxyIsoxanthohumol	Flavonoids	1.19	0.00	12.90	up
mws0028	$C_8H_8O_4$	Vanillic acid	Phenolic acids	1.19	0.01	17.13	up
mws0895	$C_{21}H_{20}O_{10}$	Genistein-7-O-Glucoside (Genistin)	Flavonoids	1.19	0.01	13.29	up
pmb2795	$C_{10}H_{10}O_3$	4-Methoxycinnamic acid	Phenolic acids	1.07	0.20	-3.05	down
MWSmce581	$C_7H_6O_3$	2,4-Dihydroxybenzaldehyde	Others	1.18	0.00	4.68	up
pmp000801	$C_{19}H_{20}O_4$	Isobavachalcone A	Flavonoids	1.19	0.01	10.09	up
pmp000802	$C_{19}H_{20}O_4$	Isobavachalcone B	Flavonoids	1.19	0.01	10.09	up
mws0921	$C_9H_{10}O_2$	p-Coumaryl alcohol	Phenolic acids	1.20	0.00	15.91	up
HJN098	$C_{42}H_{68}O_{13}$	Azukisaponin I	Terpenoids	1.20	0.00	15.86	up
Zmhn001926	$C_{13}H_{16}O_8$	1-O-Salicyloyl-β-D-glucose	Phenolic acids	1.10	0.00	1.58	up
MWSslk138	$C_7H_6O_5$	2,3,4-Trihydroxybenzoic acid	Phenolic acids	1.18	0.00	3.20	up
Zmhn002422	$C_{16}H_{20}O_9$	1-O-Feruloyl-β-D-glucose	Phenolic acids	1.18	0.00	1.49	up
pmp000086	$C_{13}H_{16}O_6$	1-Feruloyl-sn-glycerol*	Phenolic acids	1.09	0.00	2.01	up
MWSmce366	$C_{27}H_{32}O_6$	2'-Methoxykurarinone	Flavonoids	1.20	0.00	-9.20	down
Lmmp007480	$C_{15}H_{12}O_4$	2,4,4'-trihydroxychalcone	Flavonoids	1.19	0.03	6.42	up

Hmlp006326	$C_{16}H_{16}O_4$	2',4'-dihydroxy-4-methoxydihydrochalcone	Flavonoids	1.19	0.01	11.70	up
Lmbn001981	$C_7H_6O_3$	2,5-Dihydroxybenzaldehyde	Phenolic acids	1.19	0.01	3.04	up
pme3475	$C_{15}H_{12}O_5$	Butin; 7,3',4'-Trihydroxyflavanone	Flavonoids	1.20	0.00	16.87	up
pme1002	$C_8H_{11}NO$	L-Tyramine	Alkaloids	1.19	0.01	6.40	up
Lmlp005236	$C_{21}H_{22}O_{11}$	Dihydrokaempferol-3-O-glucoside	Flavonoids	1.13	0.04	2.45	up
mws1033	$C_{16}H_{14}O_6$	Homoeriodictyol	Flavonoids	1.20	0.00	14.51	up
MWStz070	$C_9H_{11}NO_3$	N-(2-Hydroxy-4-methoxyphenyl)acetamide	Alkaloids	1.15	0.04	1.09	up
Zmsp001834	$C_8H_8O_4$	2,6-Dimethoxy-1,4-benzoquinone	Quinones	1.19	0.00	3.66	up
Zmgp004060	$C_{22}H_{22}O_{11}$	paratensein-7-O-glucoside	Flavonoids	1.15	0.00	3.07	up
pmp000548	$C_{16}H_{12}O_6$	Pratensein	Flavonoids	1.19	0.00	7.02	up
Lhmp122216	$C_{15}H_{10}O_4$	nordalbergin	Lignans and Coumarins	1.18	0.05	6.56	up
Lmjp002461	$C_{27}H_{30}O_{16}$	Quercetin-3-O-neohesperidoside*	Flavonoids	1.13	0.00	-1.93	down
Lmbp002336	$C_{27}H_{30}O_{16}$	Quercetin-3-O-(2''-O-rhamnosyl)galactoside	Flavonoids	1.13	0.00	-1.93	down
mws0988	$C_{16}H_{12}O_7$	Rhamnetin; 3,5,3',4'-Tetrahydroxy-7-Methoxyflavone	Flavonoids	1.19	0.14	-12.66	down
Jmwn002117	$C_{14}H_{20}O_9$	2-(3,4-dihydroxyphenyl)ethanediol 1-O-β-D-glucopyranoside	Phenolic acids	1.19	0.01	4.18	up
pma0791	$C_{24}H_{24}O_{13}$	Naringenin-7-O-(6''-malonyl)glucoside	Flavonoids	1.18	0.00	6.76	up
Zmjp005262	$C_{16}H_{12}O_6$	2-hydroxyemodin-1-methylether	Quinones	1.18	0.00	6.04	up
NK10246260	$C_9H_7NO_2$	2,4-Dihydroxyquinoline	Alkaloids	1.01	0.00	5.38	up
Zmgn004894	$C_8H_8O_3$	Methyl 4-hydroxybenzoate	Phenolic acids	1.16	0.05	4.69	up
Smpn009074	$C_{30}H_{48}O_6$	2α,3α,19α,23-tetrahydroxy-12-ursen-28-oic acid	Terpenoids	1.19	0.00	13.73	up

mws1094	$C_{15}H_{12}O_6$	Aromadendrin (Dihydrokaempferol)	Flavonoids	1.18	0.04	6.66	up
Lmzn006169	$C_{30}H_{48}O_4$	Pomolic acid	Terpenoids	1.19	0.00	15.04	up
Lmjp003295	$C_{22}H_{22}O_{12}$	6-Methoxykaempferol-3-O-glucoside	Flavonoids	1.18	0.00	2.66	up
pmp000604	$C_{21}H_{22}O_{10}$	Rubrofusarin-6-O-glucoside	Others	1.17	0.02	2.10	up
Lmjp004941	$C_{16}H_{12}O_6$	3,5,4'-Trihydroxy-7-methoxyflavone (Rhamnocitrin)	Flavonoids	1.19	0.00	6.96	up
Lmdn009383	$C_{20}H_{18}O_5$	Glyceollin III	Flavonoids	1.19	0.02	10.80	up
Lmmn002179	$C_{14}H_{18}O_8$	Methyl salicylate-2-O-glucoside	Phenolic acids	1.16	0.00	1.60	up
pmb3041	$C_{23}H_{22}O_{14}$	Tricin-7-O-saccharic acid	Flavonoids	1.18	0.00	1.42	up
pmb2999	$C_{22}H_{22}O_{11}$	Chrysoeriol-5-O-glucoside	Flavonoids	1.13	0.00	-1.06	down
pme3261	$C_{15}H_{10}O_5$	6-Hydroxydaidzein	Flavonoids	1.17	0.02	5.52	up
Lmsn002937	$C_{22}H_{22}O_{13}$	1-O-Caffeoyl-6-O-galloyl-β-D-glucose	Phenolic acids	1.14	0.09	3.68	up
zjbp110804	$C_{20}H_{20}O_6$	6,7-dimethoxy-2-[2-(4'-hydroxy-3'-methoxyphenyl)ethyl]chromone	Others	1.19	0.00	7.57	up
Rfmb25702	$C_{28}H_{34}O_{12}$	Pinoresinol-4-O-(6"-acetyl)glucoside	Lignans and Coumarins	1.17	0.00	3.30	up
MWSmce151	$C_{20}H_{16}O_5$	Psoralidin	Lignans and Coumarins	1.19	0.01	13.59	up
pmp000344	$C_{15}H_{10}O_5$	3',4',7-Trihydroxyflavone	Flavonoids	1.18	0.03	5.33	up
Lhmp122202	$C_{16}H_{12}O_5$	stevenin	Lignans and Coumarins	1.19	0.14	16.11	up
pmp000172	$C_{23}H_{24}O_{11}$	5,2'-Dihydroxy-7,8-dimethoxyflavone glycosides*	Flavonoids	1.19	0.01	-12.43	down
HJN091	$C_{22}H_{22}O_{10}$	Prunetin-4'-O-glucoside	Flavonoids	1.19	0.07	-15.18	down
mws0183	$C_7H_6O_4$	3,4-Dihydroxybenzoic acid (Protocatechuic acid)*	Phenolic acids	1.20	0.00	16.24	up
mws1179	$C_{21}H_{22}O_{10}$	Naringenin-7-O-glucoside (Prunin)	Flavonoids	1.18	0.00	4.21	up
pmb3142	$C_{13}H_{16}O_8$	Salicylic acid-2-O-glucoside	Phenolic acids	1.18	0.00	2.23	up

mws0912	$C_{15}H_{10}O_5$	2'-Hydroxydaidzein	Flavonoids	1.19	0.12	13.76	up
pme1587	$C_{21}H_{20}O_9$	Daidzein-7-O-glucoside(Daidzin)	Flavonoids	1.02	0.03	1.77	up
Lmln001951	$C_{26}H_{28}O_{16}$	Quercetin-3-O-(6''-O-arabinosyl)glucoside	Flavonoids	1.18	0.02	-2.20	down
mad1424	$C_{17}H_{14}O_{13}$	Ditartaroyl-hydroxycoumarin	Lignans and Coumarins	1.16	0.00	1.91	up
Smgp004575	$C_{21}H_{20}O_{12}$	Quercetin-5-O-β-D-glucoside*	Flavonoids	1.13	0.01	-1.83	down
MWSslk108	$C_9H_{17}NO_4$	O-Acetyl-L-carnitine	Alkaloids	1.19	0.03	17.74	up
MWS20151	$C_{15}H_{10}O_5$	Apigenin; 4',5,7-Trihydroxyflavone	Flavonoids	1.18	0.01	6.51	up
Lmmn001294	$C_{14}H_{20}O_9$	Koaburaside	Phenolic acids	1.19	0.00	3.99	up
MWStz040	$C_5H_9NO_2$	Pterolactam	Alkaloids	1.17	0.01	1.70	up
Zmhn002301	$C_{15}H_{18}O_8$	p-Coumaric acid-4-O-glucoside*	Phenolic acids	1.19	0.00	2.44	up
pmp000607	$C_{22}H_{22}O_{12}$	1-Demethyl orange blunt-leaf casein-2-O-β-D-glucoside	Quinones	1.16	0.00	2.73	up
mws0064	$C_{15}H_{12}O_6$	Eriodictyol (5,7,3',4'-Tetrahydroxyflavanone)	Flavonoids	1.19	0.02	7.87	up
pmp001309	$C_{21}H_{20}O_{12}$	6-Hydroxykaempferol-7-O-glucoside	Flavonoids	1.17	0.00	-1.81	down
mws1389	$C_{30}H_{48}O_3$	Oleanolic acid	Terpenoids	1.19	0.01	-11.48	down
MWSHY0013	$C_{15}H_{10}O_4$	3,4'-Dihydroxyflavone	Flavonoids	1.19	0.00	5.15	up
HJAP127	$C_{33}H_{40}O_{20}$	Quercetin-3-O-(2''-O-Rhamnosyl)rutinoside	Flavonoids	1.08	0.13	-3.68	down
pmb2928	$C_{13}H_{16}O_{10}$	Gallic acid-4-O-glucoside	Phenolic acids	1.16	0.05	-2.17	down
Lmdp003286	$C_{21}H_{20}O_{12}$	Isohyperoside*	Flavonoids	1.18	0.00	-1.83	down
pma0634	$C_{28}H_{38}N_4O_4$	N1,N5-Bis(p-coumaroyl)spermine	Alkaloids	1.07	0.10	3.12	up
MWStz058	$C_{14}H_{12}N_2O_3$	7-Hydroxy-β-carboline-1-propionic acid	Alkaloids	1.01	0.01	7.46	up
pmp000356	$C_{20}H_{18}O_6$	Isolicoflavonol	Flavonoids	1.19	0.00	14.95	up
Zmgp002857	$C_{33}H_{40}O_{20}$	Quercetin-3-O-α-rhamnosyl (1→2)-[α-rhamnosyl (1→6)]-β-glucoside	Flavonoids	1.10	0.10	-3.72	down

Lmhp002800	$C_{28}H_{32}O_{17}$	2'-Hydoxy-5-methoxyGenistein-4',7-O-diglucoside	Flavonoids	1.19	0.01	5.86	up
pmb3023	$C_{21}H_{22}O_{11}$	Eriodictyol-8-C-glucoside	Flavonoids	1.19	0.00	7.86	up
mws1417	$C_9H_7NO_2$	Indole-3-carboxylic acid*	Alkaloids	1.14	0.03	1.84	up
mws0037	$C_{16}H_{12}O_4$	Formononetin (7-Hydroxy-4'-methoxyisoflavone)	Flavonoids	1.19	0.01	11.89	up
pmn001386	$C_{16}H_{16}O_4$	(3R)-Vestitol	Flavonoids	1.19	0.04	13.96	up
Hmln002355	$C_{27}H_{34}O_{12}$	5'-Methoxymatairesinoside	Lignans and Coumarins	1.15	0.00	-1.23	down
pmn001420	$C_{15}H_{18}O_9$	1-O-Caffeoyl-β-D-glucose*	Phenolic acids	1.19	0.00	2.49	up
MWSHY0138	$C_{21}H_{22}O_4$	Bavachinin	Flavonoids	1.19	0.01	13.00	up
mws0102	$C_9H_7NO_2$	Indole-5-carboxylic acid*	Alkaloids	1.14	0.00	2.13	up
Zmhn001793	$C_{15}H_{18}O_9$	6-O-Caffeoyl-D-glucose*	Phenolic acids	1.19	0.00	2.81	up
Lmtn002796	$C_{21}H_{22}O_{11}$	Aromadendrin-7-O-glucoside	Flavonoids	1.19	0.01	21.15	up
pmb0503	$C_{20}H_{30}N_4O_7$	N-(4'-O-glycosyl)-p-coumaroyl agmatine	Alkaloids	1.16	0.05	-2.57	down
mws0061	$C_{21}H_{20}O_{12}$	Quercetin-3-O-galactoside (Hyperin)	Flavonoids	1.16	0.00	-1.34	down
Hmgn004139	$C_{20}H_{22}O_7$	Isohydroxymatairesinol	Lignans and Coumarins	1.19	0.01	-12.19	down
Hmgp002148	$C_{22}H_{22}O_{12}$	Nepetin-7-O-alloside*	Flavonoids	1.19	0.01	2.42	up
mws0491	$C_8H_{11}N$	2-Phenylethylamine	Alkaloids	1.04	0.03	1.14	up
pmp000361	$C_{21}H_{22}O_5$	Licochalcone D	Flavonoids	1.20	0.00	12.62	up
pmp000194	$C_{24}H_{22}O_{13}$	6''-O-Malonylgenistin	Flavonoids	1.08	0.01	3.20	up
Zmyn003693	$C_{16}H_{12}O_4$	6-Hydroxy-2'-methoxyflavone	Flavonoids	1.19	0.00	11.08	up
pmb1912	$C_{20}H_{23}N_7O_7$	10-Formyltetrahydrofolic Acid	Alkaloids	1.19	0.00	3.46	up
pmb3066	$C_{22}H_{26}O_{12}$	5-O-p-Coumaroylshikimic acid O-glucoside	Phenolic acids	1.14	0.01	1.30	up

Lmsp004450	$C_{20}H_{22}O_6$	Dehydrodiconiferyl alcohol	Lignans and Coumarins	1.07	0.02	7.79	up
pmp000812	$C_{20}H_{20}O_6$	Leachianone G	Flavonoids	1.19	0.00	14.92	up
Wmkn002777	$C_{27}H_{30}O_{13}$	7-Hydroxy-3''-methoxy-isoflavone-7-primeveroside	Flavonoids	1.11	0.02	-1.43	down
Lmgp000659	C_8H_9NO	2-Phenylacetamide	Alkaloids	1.18	0.01	1.29	up
pmp000400	$C_{35}H_{36}O_{15}$	Licorice glycoside B	Flavonoids	1.18	0.02	5.09	up
MWSmce040	$C_8H_8O_3$	Isovanillin	Phenolic acids	1.15	0.05	4.84	up
pma0948	$C_8H_{11}NO$	Phenylethanolamine	Alkaloids	1.19	0.00	6.58	up
pmp000587	$C_{24}H_{22}O_{14}$	Luteolin-7-O-(6''-malonyl)glucoside	Flavonoids	1.14	0.07	2.04	up
pmp001310	$C_{27}H_{30}O_{17}$	6-Hydroxykaempferol-3,6-O-Diglucoside	Flavonoids	1.14	0.03	-1.36	down
pmp001311	$C_{27}H_{30}O_{17}$	6-Hydroxykaempferol-7,6-O-Diglucoside	Flavonoids	1.14	0.03	-1.36	down
Zmhp002730	$C_{27}H_{30}O_{17}$	6-Hydroxykaempferol-6,7-O-Diglucoside	Flavonoids	1.13	0.02	-1.53	down
Hmpn005101	$C_{21}H_{24}O_{11}$	Sieboldin	Flavonoids	1.19	0.00	-14.66	down
Lskp211359	$C_{20}H_{30}O_4$	6β-Hydroxyisovouacapenol C	Terpenoids	1.17	0.00	-1.77	down
Lmhn003240	$C_{11}H_{10}O_6$	Benzoylmalic acid	Phenolic acids	1.19	0.00	6.24	up
HJN005	$C_{16}H_{14}O_5$	5,2'-Dihydroxy-7-methoxyflavanone	Flavonoids	1.19	0.01	-10.48	down
Hmtn001120	$C_{14}H_{20}O_8$	5-(2-Hydroxyethyl)-2-O-glucosylphenol	Phenolic acids	1.01	0.00	6.39	up
Lmdp003110	$C_{15}H_{12}O_6$	2,6,7,4'-Tetrahydroxyisoflavanone	Flavonoids	1.19	0.00	7.46	up
Lmfp005436	$C_{27}H_{22}O_{15}$	Quercetin-3-O-(2''-O-galloyl)Arabinoside	Flavonoids	1.17	0.01	4.20	up
Zmcn004806	$C_{36}H_{36}O_{18}$	kaempferol-3-p-coumaroyldiglucoside	Flavonoids	1.18	0.04	3.60	up
Hmgn002833	$C_{20}H_{20}O_7$	4-Ketopinoresinol	Lignans and Coumarins	1.20	0.00	13.11	up
Hmln002321	$C_{24}H_{22}O_{15}$	Quercetin-3-O-(6''-O-malonyl)glucoside	Flavonoids	1.17	0.00	-5.45	down
pma0692	$C_{25}H_{31}N_3O_4$	N1,N10-Bis(p-coumaroyl)spermidine	Alkaloids	1.07	0.08	3.00	up

Lmsp003252	$C_{28}H_{32}O_{17}$	Isorhamnetin-3-O-sophoroside	Flavonoids	1.16	0.01	1.67	up
Lmmn009170	$C_{30}H_{48}O_3$	Mangiferolic acid	Terpenoids	1.19	0.01	15.53	up
pmb3012	$C_{22}H_{22}O_{11}$	Chrysoeriol-7-O-glucoside	Flavonoids	1.19	0.06	13.06	up
pmp000511	$C_{48}H_{78}O_{17}$	Kaikasaponin II	Terpenoids	1.17	0.02	6.90	up
Lmsn003582	$C_{22}H_{22}O_{12}$	1-O-Galloyl-4-O-p-Coumaroyl-β-D-glucose*	Phenolic acids	1.15	0.00	1.18	up
Hmtp000776	$C_8H_9NO_3$	4,5,6-Trihydroxy-2-cyclohexen-1-ylideneacetonitrile	Alkaloids	1.10	0.04	1.09	up
HJN090	$C_{21}H_{22}O_{10}$	Butin-7-O-glucoside	Flavonoids	1.02	0.01	1.75	up
Hmln005624	$C_{15}H_{14}O_4$	2,4,4'-trihydroxydihydrochalcone	Flavonoids	1.19	0.07	13.91	up
Lmmn003398	$C_{23}H_{22}O_{12}$	Kaempferol-3-O-(6"-O-acetyl)glucoside	Flavonoids	1.01	0.15	2.75	up
Lmmp004504	$C_{15}H_{10}O_6$	2'-Hydroxygenistein	Flavonoids	1.19	0.02	13.02	up
pma6460	$C_{16}H_{18}O_8$	4-O-p-Coumaroylquinic acid	Phenolic acids	1.18	0.01	-2.38	down
MWSHY0015	$C_{15}H_{12}O_4$	Liquiritigenin	Flavonoids	1.19	0.01	17.06	up
Hmgp001888	$C_{28}H_{32}O_{17}$	Patuletin-3-O-rutinoside	Flavonoids	1.19	0.02	6.05	up
HJN087	$C_{21}H_{22}O_{10}$	Naringenin-4'-O-glucoside	Flavonoids	1.17	0.00	1.05	up
Lmyn001269	$C_{27}H_{30}O_{16}$	Kaempferol-3-O-sophoroside	Flavonoids	1.03	0.02	1.49	up
Lmyn003971	$C_{26}H_{34}O_{11}$	Dihydrodehydrodiconiferyl alcohol-4-O-glucoside	Lignans and Coumarins	1.19	0.02	-11.82	down
Lmmn002274	$C_{26}H_{34}O_{11}$	Isolariciresinol-9'-O-glucoside	Lignans and Coumarins	1.19	0.02	-11.82	down
pme3233	$C_{16}H_{12}O_5$	Calycosin	Flavonoids	1.19	0.10	15.90	up
Lmmp009194	$C_{21}H_{24}O_4$	Amorfrutin A	Phenolic acids	1.19	0.00	-8.41	down
mws0025	$C_6H_6O_3$	Pyrogallol	Phenolic acids	1.19	0.10	-12.53	down
Hmgp002036	$C_{22}H_{22}O_{12}$	Nepetin-7-O-glucoside(Nepitrin)*	Flavonoids	1.19	0.00	2.49	up
pmp000193	$C_{24}H_{22}O_{12}$	6"-O-Malonyldaidzin	Flavonoids	1.16	0.01	5.41	up
mws1172	$C_{22}H_{22}O_{10}$	Trifolirhizin (Maackiain-3-O-glucoside)	Flavonoids	1.19	0.05	-14.20	down
pmp000414	$C_{27}H_{30}O_{14}$	Puerarin-4'-O-glucoside	Flavonoids	1.12	0.01	4.20	up

Lmjp002596	$C_{26}H_{28}O_{16}$	Quercetin-3-O-sambubioside*	Flavonoids	1.12	0.05	-2.41	down
MWSslk163	$C_{20}H_{20}O_4$	Isobavachin	Flavonoids	1.19	0.04	12.23	up
Lmmp008478	$C_{48}H_{76}O_{18}$	Soyasapogenol E-3-O-rhamnosyl(1,2)glucosyl (1,2)glucuronide	Terpenoids	1.19	0.07	9.65	up
pmb0680	$C_{36}H_{36}O_{17}$	Vitexin-7-O-(6''-p-coumaroyl)glucoside	Flavonoids	1.02	0.00	5.68	up
Lmmp005689	$C_{16}H_{12}O_6$	Cajanin	Flavonoids	1.19	0.01	7.07	up
pmb0608	$C_{25}H_{24}O_{14}$	Chrysoeriol-7-O-(6''-malonyl)glucoside	Flavonoids	1.19	0.00	3.18	up
pme3081	$C_8H_{12}N_2O_2$	4-(Aminomethyl)-5-(hydroxymethyl)-2-methylpyridin-3-ol	Alkaloids	1.18	0.00	1.35	up
pmb0501	$C_5H_{14}N_4$	Agmatine	Alkaloids	1.17	0.02	1.51	up
pme2024	$C_{10}H_{12}N_2O$	Serotonin	Alkaloids	1.19	0.01	12.30	up
Hmbn005456	$C_{21}H_{20}O_8$	1-O-Caffeoyl-3-O-p-coumaroylglycerol	Phenolic acids	1.20	0.00	-11.38	down
MWSmce247	$C_8H_8O_2$	2-Methylbenzoic acid	Phenolic acids	1.19	0.00	10.28	up
pmb0426	$C_{13}H_{18}N_2O$	N,N-Dimethyl-5-methoxytryptamine	Alkaloids	1.19	0.01	6.91	up
Hmgp002080	$C_{28}H_{32}O_{17}$	Patuletin-7-O-rutinoside	Flavonoids	1.19	0.01	-14.60	down
pme2831	$C_4H_{11}N_5$	1,1-Dimethylbiguanide	Alkaloids	1.19	0.01	-10.77	down
mws0856	$C_{21}H_{20}O_{12}$	Quercetin-4'-O-glucoside (Spiraeoside)	Flavonoids	1.15	0.00	-1.44	down
Cmsp003083	$C_{26}H_{32}O_{11}$	Dehydrodiconiferyl alcohol-4-O-glucoside	Lignans and Coumarins	1.19	0.01	6.57	up
mws0062	$C_{15}H_{10}O_6$	Isoluteolin (Orobol)(5,7,3',4'-tetrahydroxyisoflavone)	Flavonoids	1.19	0.02	4.74	up
pmb3072	$C_{22}H_{26}O_{12}$	3-O-p-Coumaroylshikimic acid-O-glucoside	Phenolic acids	1.18	0.00	2.84	up
pmn001421	$C_{16}H_{18}O_8$	3-O-p-Coumaroylquinic acid*	Phenolic acids	1.17	0.03	-2.38	down
Lmmp002995	$C_{30}H_{32}O_{20}$	Quercetin-7-O-(2''-malonyl)glucosyl-5-O-glucoside	Flavonoids	1.19	0.01	3.89	up
pmp000589	$C_{24}H_{22}O_{15}$	Quercetin-7-O-(6''-malonyl)glucoside	Flavonoids	1.18	0.00	-1.37	down

MWSHY0067	$C_{27}H_{30}O_{16}$	Quercetin-3-O-rutinoside (Rutin)*	Flavonoids	1.14	0.01	-2.31	down
pma0702	$C_{29}H_{39}N_3O_8$	N1,N8-Bis(sinapoyl)spermidine	Alkaloids	1.17	0.00	3.50	up
MWSHY0107	$C_{20}H_{20}O_4$	Bavachin	Flavonoids	1.18	0.19	9.38	up
MWSmce460	C_5H_9NO	2-Piperidone	Alkaloids	1.17	0.02	1.57	up
Hmcp001489	$C_{34}H_{42}O_{22}$	Isorhamnetin-3-O-(2"-O-glucosyl)galactoside-7-O-glucoside	Flavonoids	1.20	0.00	12.43	up
Lmhp005784	$C_{24}H_{28}N_2O_5$	p-Coumaroylferuloylcadaverine	Alkaloids	1.19	0.01	17.13	up
MWS20194	$C_9H_8O_2$	Cinnamic acid	Phenolic acids	1.19	0.01	-12.25	down
pme3443	$C_{11}H_{12}O_4$	Sinapinaldehyde	Phenolic acids	1.19	0.01	13.35	up
Hmlp005443	$C_{15}H_{10}O_4$	6,7-Dihydroxyflavone	Flavonoids	1.20	0.00	16.37	up
MWSHY0016	$C_{21}H_{20}O_{11}$	Luteolin-6-C-glucoside (Isoorientin)	Flavonoids	1.02	0.00	5.66	up
pmp000201	$C_{48}H_{78}O_{18}$	Soyasaponin βb (Soyasaponin I)	Terpenoids	1.19	0.06	11.72	up
pmb0484	$C_5H_{14}NO^+$	Choline	Alkaloids	1.17	0.00	-1.34	down
Hmgp002189	$C_{22}H_{22}O_{11}$	Hispidulin-7-O-glucoside(Homoplantaginin)	Flavonoids	1.13	0.00	3.05	up
MWSHY0058	$C_{15}H_{10}O_6$	Luteolin (5,7,3',4'-Tetrahydroxyflavone)	Flavonoids	1.19	0.00	13.07	up
pmp000417	$C_{21}H_{20}O_9$	Daidzein-4'-O-glucoside	Flavonoids	1.18	0.00	3.93	up
pmn001642	$C_{23}H_{20}O_{13}$	Kaempferol-3-O-(2"-O-acetyl)glucuronide	Flavonoids	1.19	0.00	-1.36	down
HJAP023	$C_{33}H_{40}O_{21}$	Kaempferol-6,8-di-C-glucoside-7-O-glucoside	Flavonoids	1.19	0.01	12.37	up
pmp000343	$C_{16}H_{12}O_4$	Isoformononetin	Flavonoids	1.07	0.00	8.41	up
MWS20177	$C_{15}H_8O_5$	5-hydroxy-anthraquinone-2-carboxylic acid	Quinones	1.20	0.00	11.40	up
Hmbn002692	$C_{22}H_{30}O_{14}$	6'-O-Feruloyl-D-sucrose	Phenolic acids	1.19	0.02	15.05	up
Lmsn004162	$C_{22}H_{22}O_{12}$	1-O-Galloyl-2-O-p-Coumaroyl-β-D-glucose	Phenolic acids	1.19	0.11	-10.68	down

Lmmn004625	$C_{21}H_{22}O_{11}$	Dihydrokaempferol-7-O-glucoside	Flavonoids	1.19	0.00	15.32	up
Cmzp002057	$C_{14}H_{18}O_8$	6-O-Acetylarbutin	Phenolic acids	1.20	0.00	11.63	up
HJN086	$C_{21}H_{22}O_{11}$	Eriodictyol-3'-O-glucoside	Flavonoids	1.19	0.00	15.73	up
MWSmce588	$C_{16}H_{14}O_5$	Licochalcone B	Flavonoids	1.18	0.00	-2.25	down
HJN003	$C_{17}H_{22}O_{10}$	1-O-Sinapoyl-β-D-glucose	Phenolic acids	1.16	0.00	2.54	up
Lmjp003655	$C_{22}H_{22}O_{11}$	6-C-MethylKaempferol-3-glucoside	Flavonoids	1.16	0.00	3.10	up
pmn001551	$C_{21}H_{22}O_9$	Natsudaidain (3-Hydroxy-3',4',5,6,7,8-hexamethoxyflavone)	Flavonoids	1.20	0.00	11.08	up
pmb0764	C_6H_9NOS	4-Methyl-5-thiazoleethanol	Others	1.18	0.19	-13.42	down
pme2693	$C_6H_{14}N_2O$	N-Acetylputrescine	Alkaloids	1.18	0.00	1.34	up
Lmjn005433	$C_{19}H_{18}O_8$	Rosmarinic acid methyl ester	Phenolic acids	1.19	0.00	13.31	up
mws4060	$C_{16}H_{14}O_4$	Echinatin	Flavonoids	1.19	0.05	12.70	up
pmp000627	$C_{15}H_{20}N_2O_2$	Isobaptifoline	Alkaloids	1.19	0.00	10.54	up
Hmyn001360	$C_{18}H_{20}O_4$	Doitungbiphenyl A	Phenolic acids	1.15	0.00	1.66	up
Hmcp002207	$C_{22}H_{22}O_{12}$	Isorhamnetin-7-O-glucoside (Brassicin)*	Flavonoids	1.18	0.00	2.72	up
mws4061	$C_{21}H_{22}O_4$	Licochalcone A	Flavonoids	1.20	0.00	-11.33	down
MWSHY0081	$C_{21}H_{22}O_4$	Licochalcone E	Flavonoids	1.20	0.00	-11.33	down
pmb0660	$C_{36}H_{36}O_{18}$	Isoorientin-7-O-(6''-p-coumaroyl)glucoside	Flavonoids	1.18	0.00	2.40	up
MWSmce577	$C_{12}H_{14}O_3$	(E)-Ethyl p-methoxycinnamate	Phenolic acids	1.19	0.01	11.42	up
MWS0687	$C_7H_8N_2O_2$	1-Methyl-6-Oxo-1,6-Dihydropyridine-3-Carboxamide	Alkaloids	1.19	0.03	-13.55	down
pma2088	$C_{27}H_{30}O_{17}$	Quercetin-3,4'-O-di-glucoside	Flavonoids	1.19	0.01	11.93	up
GQ512001	$C_{33}H_{40}O_{21}$	Quercetin-3-O-glucosyl(1→3)rhamnosyl(1→6)Galactoside	Flavonoids	1.19	0.13	11.89	up
Lmjp003206	$C_{32}H_{38}O_{21}$	Quercetin-3-O-Sambubioside-5-O-Glucoside	Flavonoids	1.13	0.03	-1.79	down

Hmmp004965	$C_{22}H_{22}O_{11}$	Diosmetin-7-O-glucoside*	Flavonoids	1.15	0.00	2.99	up
MWSslk232	$C_{28}H_{32}O_{16}$	Complanatuside	Flavonoids	1.10	0.01	-1.95	down
pmb3014	$C_{31}H_{30}O_{15}$	Luteolin-7-O-(6"-eudesmyl)glucoside	Flavonoids	1.19	0.03	14.08	up
MWSHY0112	$C_{21}H_{22}O_{5}$	Xanthohumol	Flavonoids	1.16	0.00	-1.62	down
pmb3075	$C_{16}H_{16}O_{7}$	3-O-p-Coumaroylshikimic acid	Phenolic acids	1.05	0.09	-1.04	down
Zmxp005470	$C_{26}H_{26}O_{15}$	Tricin-7-O-(6"-O-malonyl)glucoside	Flavonoids	1.19	0.01	-12.24	down
Wmkn004416	$C_{20}H_{20}O_{5}$	Desmethylxanthohumol	Flavonoids	1.19	0.01	10.44	up
Hmgp001996	$C_{15}H_{10}O_{8}$	Quercetagetin; 3,3',4',5,6,7-Hexahydroxyflavone	Flavonoids	1.02	0.02	7.03	up
Lmmn004032	$C_{11}H_{16}O$	Cis-Jasmone	Others	1.19	0.00	5.63	up
Lmyn002403	$C_{14}H_{18}O_{8}$	Mandelic acid-β-glucoside	Phenolic acids	1.15	0.00	1.51	up
Hmcn006267	$C_{9}H_{10}O_{4}$	2,6-Dimethoxybenzoic acid	Phenolic acids	1.19	0.01	11.72	up
MWSmce455	$C_{9}H_{10}O_{4}$	Vanillic acid methyl ester	Phenolic acids	1.19	0.01	11.72	up
Cmbp005948	$C_{21}H_{20}O_{8}$	1-O-p-Hydroxycinnamoyl-3-O-caffeoylglycerol	Phenolic acids	1.18	0.02	-1.90	down
Zmsp004363	$C_{23}H_{22}O_{13}$	Quercetin-3-O-(6"-O-acetyl)glucoside	Flavonoids	1.19	0.17	-11.96	down
Zmdp004370	$C_{23}H_{22}O_{10}$	6"-O-Acetyldaidzin	Flavonoids	1.19	0.04	12.26	up
Hmbn005207	$C_{30}H_{48}O_{4}$	Hederagenin	Terpenoids	1.19	0.01	14.07	up
Lmtn002565	$C_{14}H_{18}O_{9}$	1-O-Vanilloyl-D-Glucose	Phenolic acids	1.19	0.02	14.56	up
Lmjp003589	$C_{35}H_{40}O_{23}$	Kaempferol-3-O-(2"-apiosyl-4"-glucosyl-6"-malonyl)Glucoside	Flavonoids	1.16	0.00	2.76	up
Zmbp008419	$C_{20}H_{20}O_{5}$	6-prenylnaringenin	Flavonoids	1.20	0.00	14.50	up
MWSHY0046	$C_{21}H_{20}O_{12}$	Quercetin-3-O-glucoside (Isoquercitrin)*	Flavonoids	1.17	0.00	-1.87	down
Zmhn001883	$C_{14}H_{18}O_{9}$	Vanillic acid-4-O-glucoside	Phenolic acids	1.19	0.02	15.10	up
pmb0711	$C_{27}H_{30}O_{16}$	Quercetin-7-O-rutinoside	Flavonoids	1.16	0.02	-2.44	down

Lmap001823	$C_{14}H_{17}NO_7$	Dhurrin	Alkaloids	1.17	0.00	-1.34	down
pmp000240	$C_{27}H_{28}O_{16}$	Apigenin-6-C-(2"-glucuronyl)glucoside	Flavonoids	1.19	0.01	12.56	up
Lmhn003246	$C_{24}H_{22}O_{13}$	Sinapoylcaffeoyltartaric acid	Phenolic acids	1.19	0.00	11.05	up
pmp000348	$C_{20}H_{18}O_4$	Kanzonol D	Flavonoids	1.19	0.00	11.96	up
Lmmp003266	$C_{29}H_{30}O_{19}$	Quercetin-3-O-(2"-O-malonyl)glucoside-7-O-arabinoside	Flavonoids	1.17	0.00	-3.66	down
Zmhp003322	$C_{36}H_{36}O_{17}$	Isovitexin-2"-O-(6'''-p-coumaroyl)glucoside	Flavonoids	1.05	0.00	6.68	up
MWSmce177	$C_{12}H_{14}O_4$	Ethyl ferulate	Phenolic acids	1.19	0.03	13.19	up
pmp000003	$C_{16}H_{12}O_7$	Nepetin (5,7,3',4'-Tetrahydroxy-6-methoxyflavone)	Flavonoids	1.19	0.11	11.26	up
mws4053	$C_{30}H_{48}O_3$	Ursolic acid	Terpenoids	1.04	0.00	6.83	up
HJAP148	$C_{26}H_{28}O_{15}$	Kaempferol-3-O-sambubioside	Flavonoids	1.19	0.04	-13.04	down
pme3504	$C_{22}H_{22}O_9$	Formononetin-7-O-glucoside (Ononin)	Flavonoids	1.12	0.00	-2.84	down
MWSmce338	$C_6H_{11}NO_3$	N-Hydroxypipecolic acid	Alkaloids	1.18	0.02	-1.52	down
Lmhp003658	$C_{27}H_{28}O_{14}$	Monohydroxy-trimethoxyflavone-O-(6"-malonyl)glucoside	Flavonoids	1.04	0.01	1.41	up
Lnrp102163	$C_{33}H_{40}O_{20}$	Quercetin-3-O-rutinoside-7-O-rhamnoside	Flavonoids	1.12	0.03	-1.60	down
MWSmce221	$C_{10}H_8O_4$	5,7-Dihydroxy-4-methylcoumarin	Lignans and Coumarins	1.19	0.01	-13.22	down
Lmwp004293	$C_{42}H_{46}O_{24}$	Quercetin-3-O-(2'''-O-p-coumaroyl)sophoroside-7-O-glucoside	Flavonoids	1.17	0.01	-1.86	down
pmn001704	$C_{30}H_{46}O_4$	2,3-Dihydroxy-5(6),12(13)-diene-ursolic acid	Terpenoids	1.19	0.02	8.04	up
Lmdn003756	$C_{10}H_{10}O_4$	Methyl caffeate	Phenolic acids	1.19	0.03	16.09	up
MWSmce653	$C_9H_{10}O_2$	2'-Hydroxy-4'-Methylacetophenone	Phenolic acids	1.19	0.01	11.01	up
MWSHY0164	$C_{20}H_{18}O_4$	Licoflavone A	Flavonoids	1.19	0.03	11.90	up
pmn001710	$C_{24}H_{26}O_{13}$	Rosmarinic acid-3'-O-glucoside	Phenolic acids	1.19	0.00	4.46	up

mws0885	$C_7H_6O_4$	2,4-Dihydroxybenzoic acid	Phenolic acids	1.19	0.01	16.35	up
pmp000199	$C_{48}H_{74}O_{17}$	Soyasaponin γg	Terpenoids	1.19	0.05	8.65	up
Lmtp004044	$C_{26}H_{28}O_{16}$	Quercetin-3-O-apiosyl(1→2)galactoside*	Flavonoids	1.11	0.03	-2.15	down
Lmfp001509	$C_{13}H_{16}O_9$	1-O-Galloyl-rhamnose	Phenolic acids	1.19	0.01	12.38	up
Zmln000899	$C_{15}H_{20}O_9$	Syringaldehyde-4-O-glucoside	Phenolic acids	1.16	0.01	1.28	up
pmb2991	$C_{33}H_{40}O_{19}$	Apigenin-7-O-glucoside-4'-O-rutinoside	Flavonoids	1.18	0.01	3.50	up
MWSslk006	$C_{10}H_{12}O_4$	Ethyl Vanillate	Phenolic acids	1.19	0.03	-10.03	down
Hmcp002316	$C_{21}H_{20}O_{11}$	Isorhamnetin-3-O-arabinoside	Flavonoids	1.19	0.03	12.75	up
pmp000645	$C_{26}H_{30}O_5$	Kushenol U	Flavonoids	1.12	0.01	-1.31	down
Lmmn002260	$C_{27}H_{36}O_{12}$	5'-Methoxyisolariciresinol-9'-O-glucoside	Lignans and Coumarins	1.19	0.01	3.54	up
Hmln002199	$C_{23}H_{22}O_{13}$	Quercetin-3-O-(6''-O-acetyl)galactoside	Flavonoids	1.16	0.00	-1.59	down
pma3649	$C_5H_9NO_3$	5-Aminolevulinic Acid	Alkaloids	1.19	0.00	2.41	up
HJN104	$C_{21}H_{22}O_{13}$	Dihydromyricetin-3-O-glucoside	Flavonoids	1.19	0.01	11.60	up
pmp000195	$C_{25}H_{24}O_{13}$	6''-O-Malonylglycitin	Flavonoids	1.19	0.03	4.36	up
Hmcp001618	$C_{32}H_{38}O_{20}$	Quercetin-3-O-(2''-O-Xylosyl)rutinoside	Flavonoids	1.18	0.01	1.74	up
Lmgp002595	$C_9H_{10}O_3$	4'-Hydroxy-3'-methoxyacetophenone (Acetovanillone)	Phenolic acids	1.19	0.01	15.60	up
pmb0645	$C_{28}H_{34}O_{16}$	Hesperetin-6-C-glucoside-7-O-glucoside	Flavonoids	1.13	0.00	-1.55	down
pmb0492	$C_{34}H_{37}N_3O_6$	N',N'',N'''-p-Coumaroyl-cinnamoyl-caffeoyl spermidine	Alkaloids	1.10	0.01	1.69	up
HJAP056	$C_{23}H_{24}O_{11}$	Dihydroxy-dimethoxyflavone-7-O-glucoside*	Flavonoids	1.19	0.00	-12.41	down
Rfmb26201	$C_{30}H_{38}O_{14}$	Syringaresinol-4'-O-(6''-acetyl)glucoside	Lignans and Coumarins	1.10	0.03	1.99	up

pma0724	$C_{21}H_{22}O_{10}$	Naringenin-6-C-Glucoside	Flavonoids	1.14	0.00	2.04	up
Lmmp003306	$C_{25}H_{26}O_{15}$	Quercetin-3-O-xylosyl(1→2)arabinoside	Flavonoids	1.17	0.03	-2.90	down
Cmbn007148	$C_{22}H_{22}O_9$	1-O-Feruloyl-3-O-caffeoylglycerol	Phenolic acids	1.01	0.09	-1.58	down
Lmdp004668	$C_{37}H_{38}O_{19}$	Kaempferol-3-O-(6''-Feruloyl)glucosyl-(1→4)-galactoside	Flavonoids	1.19	0.00	3.33	up
MWSslk066	$C_8H_8O_4$	3-Hydroxy-4-methoxybenzoic acid; Isovanillic Acid	Phenolic acids	1.19	0.00	14.82	up
Lmzp004885	$C_{17}H_{14}O_7$	Tricin (5,7,4'-Trihydroxy-3',5'-dimethoxyflavone)	Flavonoids	1.01	0.01	5.48	up
pme1292	$C_8H_8O_4$	Homogentisic acid	Phenolic acids	1.20	0.00	-15.16	down
mws0072	$C_{21}H_{20}O_{10}$	Apigenin-5-O-glucoside	Flavonoids	1.14	0.00	1.85	up
pmb0508	$C_{14}H_{20}N_4O_2$	p-Coumaroylagmatine	Alkaloids	1.19	0.09	12.46	up
Hmdp007781	$C_{22}H_{18}O_4$	1-p-Hydroxybenzyl-4-methoxy phenanthrene-2,7-diol	Quinones	1.19	0.03	-11.78	down
Lmbp006165	$C_{17}H_{18}O_4$	6,7-cis-Dihydroxy-2-(2-phenylethyl)-5,6,7,8-tetrahydrochromone	Others	1.18	0.01	-2.69	down
Lmfn001344	$C_{20}H_{20}O_{13}$	Maplexin C (2,3-Di-O-Galloyl-1,5-Anhydro-D-Glucitol)	Phenolic acids	1.12	0.02	2.35	up
Lmmp002755	$C_{33}H_{40}O_{21}$	Quercetin-7-O-rutinoside-4'-O-glucoside	Flavonoids	1.12	0.02	-1.98	down
Lmbp003825	$C_{15}H_{16}O_8$	Skimmin (7-Hydroxycoumarin-7-O-glucoside)	Lignans and Coumarins	1.19	0.03	13.54	up
pmn001375	$C_{26}H_{32}O_{12}$	1-Hydroxypinoresinol-1-O-Glucoside	Lignans and Coumarins	1.20	0.00	12.42	up
Hmsp005265	$C_{16}H_{16}O_5$	3'-Deoxysappanol	Others	1.19	0.05	13.65	up
pmp000358	$C_{21}H_{22}O_5$	Licoagrochalcone D	Flavonoids	1.19	0.01	12.38	up
pmn001370	$C_{32}H_{42}O_{16}$	Pinoresinol-4,4'-O-di-O-glucoside	Lignans and Coumarins	1.18	0.00	4.56	up
Lmdp005678	$C_{42}H_{68}O_{14}$	Soyasaponin III	Terpenoids	1.19	0.13	9.45	up

MWSHY0140	$C_{16}H_{12}O_4$	3-Hydroxy-3'-methoxyflavone	Flavonoids	1.19	0.09	10.86	up
Lmqp002170	$C_{33}H_{40}O_{21}$	Kaempferol-3-O-sophorotrioside	Flavonoids	1.16	0.00	4.16	up
Lhmp122104	$C_{29}H_{24}O_{10}$	Diferuloyl caffeic acid	Others	1.14	0.00	1.80	up
pmp001245	$C_{15}H_{22}NO_4^+$	Feruloylcholine	Alkaloids	1.19	0.09	11.56	up
Lmqn001795	$C_{16}H_{12}O_4$	5-Hydroxy-7-methoxyflavone	Flavonoids	1.19	0.00	11.78	up
MWS1844	$C_{14}H_{18}O$	2-Pentyl-3-phenyl-2-propenal	Others	1.19	0.01	8.45	up
pmp000413	$C_{21}H_{20}O_{10}$	Genistein-8-C-glucoside	Flavonoids	1.01	0.13	2.41	up
MWSmce283	$C_9H_{10}O_2$	4'-Hydroxypropiophenone	Phenolic acids	1.19	0.00	11.65	up
Lmbn004790	$C_{11}H_{14}O_4$	Methyl 3-(3-hydroxy-4-methoxyphenyl)propanoate	Phenolic acids	1.19	0.01	13.22	up
Zmdp004305	$C_{28}H_{24}O_9$	Daidzein-7-O-(2''-benzoyl)rhamnoside	Flavonoids	1.18	0.00	4.12	up
pmp000357	$C_{20}H_{18}O_6$	Licoisoflavanone	Flavonoids	1.19	0.01	11.64	up
MWSmce108	$C_{15}H_{12}O_5$	Butein	Flavonoids	1.19	0.02	12.44	up
Lmsp002982	$C_{33}H_{40}O_{21}$	Quercetin-3-O-sophoroside-7-O-rhamnoside	Flavonoids	1.19	0.02	-13.09	down
Lmmp002334	$C_{33}H_{40}O_{21}$	Quercetin-3-O-rutinoside-7-O-glucoside	Flavonoids	1.19	0.02	-13.09	down
Hmcp001329	$C_{32}H_{38}O_{21}$	Quercetin-3-O-xylosyl(1→2)glucosyl(1→2)glucoside	Flavonoids	1.19	0.01	10.84	up
pmb0626	$C_{33}H_{40}O_{20}$	Apigenin-6-C-glucoside-7-O-Sophoroside	Flavonoids	1.19	0.03	-11.66	down
Li512113	$C_{20}H_{20}O_{12}$	Maleoyl-caffeoylquinic acid	Phenolic acids	1.19	0.01	10.64	up
Hmyp002656	$C_{11}H_{11}NO_4$	Methyl dioxindole-3-acetate	Alkaloids	1.03	0.06	1.37	up
pmp000585	$C_{24}H_{22}O_{13}$	Apigenin-7-O-(6''-malonyl)glucoside	Flavonoids	1.16	0.00	2.80	up
pma6640	$C_{22}H_{22}O_{12}$	3'-O-Methyltricetin-7-O-glucoside	Flavonoids	1.19	0.00	4.67	up

Lmdp004461	$C_{43}H_{48}O_{24}$	Quercetin-3-O-(6''-O-feruloyl)glucoside-7-O-rutinoside	Flavonoids	1.14	0.00	1.98	up
Lmmp002560	$C_{27}H_{28}O_{18}$	Quercetin-3-O-(2''-O-glucosyl)glucuronide	Flavonoids	1.12	0.02	-1.71	down
mws0180	$C_7H_6O_4$	2,5-Dihydroxybenzoic acid; Gentisic Acid*	Phenolic acids	1.20	0.00	14.95	up
pme2244	$C_{11}H_{11}NO_2$	3-Indolepropionic acid	Alkaloids	1.19	0.04	11.04	up
pmp000393	$C_{26}H_{30}O_{13}$	Isoliquiritin apioside	Flavonoids	1.16	0.04	2.91	up
pmp000392	$C_{26}H_{30}O_{13}$	Liquiritigenin-7-O-apioside-4'-O-glucoside	Flavonoids	1.16	0.04	2.91	up
mws4114	$C_{20}H_{18}O_4$	3,4-Didehydroglabridin	Flavonoids	1.19	0.01	10.88	up
mws0853	$C_{11}H_{14}O_4$	Sinapyl alcohol	Phenolic acids	1.19	0.15	12.53	up
MWSHY0029	$C_{15}H_{10}O_7$	Quercetin	Flavonoids	1.19	0.13	11.75	up
mws0566	$C_7H_8O_2$	4-Methylcatechol	Phenolic acids	1.19	0.01	11.36	up
pmp000598	$C_{16}H_{12}O_5$	Obtusifolin	Quinones	1.19	0.07	11.16	up
HJAP061	$C_{32}H_{38}O_{20}$	Quercetin-3-O-(2''-O-arabinosyl)rutinoside	Flavonoids	1.18	0.03	-3.15	down
mws0009	$C_{10}H_{10}O_3$	Coniferaldehyde	Phenolic acids	1.16	0.00	3.41	up
MWS20148	$C_{21}H_{20}O_{10}$	Apigenin-4'-O-glucoside*	Flavonoids	1.14	0.00	3.43	up
MWS20149	$C_{21}H_{20}O_{10}$	Apigenin-7-O-glucoside(Cosmosiin)*	Flavonoids	1.14	0.00	3.43	up
Li512111	$C_{24}H_{24}O_{13}$	Isorhamnetin-3-O-(6''-acetylglucoside)	Flavonoids	1.19	0.03	13.68	up
pmp000360	$C_{21}H_{22}O_5$	3-Hydroxylicochalcone A	Flavonoids	1.19	0.02	11.81	up
pma6353	$C_{21}H_{20}O_9$	Chrysin-8-C-glucoside	Flavonoids	1.16	0.04	3.89	up
Hmmp008597	$C_{16}H_{14}O_4$	4,4'-Dihydroxy-2'-methoxychalcone (3-Deoxysappanchalcone)	Flavonoids	1.18	0.14	-10.70	down
pmb0819	$C_{10}H_8N_2$	3-Indoleacetonitrile	Alkaloids	1.12	0.03	1.29	up
Lmjp003360	$C_{35}H_{40}O_{24}$	Quercetin-3-O-(2''-O-malonyl)sophoroside-7-O-arabinoside	Flavonoids	1.07	0.02	1.96	up
Lmgn001670	$C_7H_6O_3$	Salicylic acid	Phenolic acids	1.12	0.05	2.71	up
pmp000087	$C_{13}H_{16}O_6$	2-Feruloyl-sn-glycerol*	Phenolic acids	1.20	0.00	13.64	up

Lmyp003951	$C_{11}H_{14}O_5$	3-Hydroxy-1-(4-hydroxy-3,5-dimethoxyphenyl)propan-1-one	Phenolic acids	1.18	0.00	3.20	up
pma1840	$C_{14}H_{20}NO_3^+$	4-Coumaroylcholine	Alkaloids	1.19	0.18	-14.57	down
Lmgp001898	$C_9H_7NO_2$	4,6-Dihydroxyquinoline	Alkaloids	1.19	0.01	12.38	up
Zmhp006502	$C_{25}H_{24}O_{14}$	Kaempferide-3-O-(6"-malonyl)glucoside	Flavonoids	1.19	0.00	-12.36	down
MWSmce064	$C_{16}H_{14}O_6$	Hematoxylin	Phenolic acids	1.19	0.06	-10.10	down
MWSslk164	$C_{20}H_{18}O_4$	Neobavaisoflavone	Flavonoids	1.19	0.00	14.09	up
Cwjp002681	$C_{42}H_{46}O_{23}$	Kaempferol-3-O-(2'''-p-Coumaroyl)sophoroside-7-O-Glucoside	Flavonoids	1.15	0.00	2.39	up
MWS1830	$C_9H_{10}O_3$	Ethylsalicylate	Phenolic acids	1.19	0.00	-10.60	down
Lmbp002592	$C_{27}H_{30}O_{16}$	Kaempferol-3,7-O-diglucoside	Flavonoids	1.14	0.00	2.13	up
HJN041	$C_{21}H_{24}O_{11}$	Epicatechin glucoside	Flavonoids	1.19	0.13	12.98	up
pma0214	$C_{22}H_{22}O_{12}$	6-C-Methylquercetin-3-O-glucoside	Flavonoids	1.02	0.02	6.19	up
Zmdp004112	$C_{24}H_{22}O_{13}$	Genistein-7-O-(6"-malonyl)glucoside	Flavonoids	1.08	0.00	2.46	up
pmb0824	$C_{25}H_{28}O_{13}$	Syringic acid-4-O-(6"-feruloyl)glucoside	Phenolic acids	1.19	0.18	11.04	up
mws0027	$C_9H_{10}O_5$	Syringic acid	Phenolic acids	1.19	0.01	12.91	up
Zmdp003228	$C_{26}H_{28}O_{13}$	Daidzein-7-O-apiosyl(1→6)glucoside	Flavonoids	1.12	0.01	3.05	up
Zmhp005139	$C_{25}H_{24}O_{15}$	Tamarixetin-3-O-(6"-malonyl)glucoside	Flavonoids	1.15	0.02	-1.07	down
Hmcp002187	$C_{23}H_{24}O_{13}$	Limocitrin-3-O-galactoside	Flavonoids	1.01	0.03	4.88	up
Hmcp002399	$C_{23}H_{24}O_{13}$	Limocitrin-3-O-glucoside	Flavonoids	1.01	0.03	4.88	up
pmp000241	$C_{27}H_{28}O_{17}$	Luteolin-6-C-(2"-glucuronyl)glucoside	Flavonoids	1.19	0.01	9.87	up
pmp000368	$C_{22}H_{22}O_6$	Licoricone	Flavonoids	1.19	0.01	10.84	up
Lmbp003668	$C_{20}H_{18}O_{10}$	Kaempferol-3-O-arabinoside	Flavonoids	1.19	0.02	11.01	up
pmp000200	$C_{48}H_{76}O_{18}$	Soyasaponin βe	Terpenoids	1.19	0.03	9.58	up

Lmsn005253	$C_{29}H_{26}O_{15}$	1,2-Di-O-galloyl-3-O-Cinnamoyl-β-D-glucose	Phenolic acids	1.19	0.02	-12.23	down
Lmsn005877	$C_{29}H_{26}O_{15}$	1,2-Di-O-galloyl-4-O-Cinnamoyl-β-D-glucose	Phenolic acids	1.19	0.02	-12.23	down
pmb0667	$C_{36}H_{36}O_{18}$	Isoorientin-7-O-(6''-feruloyl)arabinoside	Flavonoids	1.03	0.01	6.03	up
Zmjp004852	$C_{23}H_{24}O_{13}$	5,6,3',4'-Tetrahydroxy-3,7-dimethoxyflavone-6-O-glucoside	Flavonoids	1.19	0.01	11.80	up

The secondary metabolites with the variable importance in projection (VIP) to ≥1 and log2 |fold-change|≥1 were selected.
